# Evaluation of a Decision Support System for Obstructive Sleep Apnea with Nonlinear Analysis of Respiratory Signals

**DOI:** 10.1371/journal.pone.0150163

**Published:** 2016-03-03

**Authors:** Evangelos Kaimakamis, Venetia Tsara, Charalambos Bratsas, Lazaros Sichletidis, Charalambos Karvounis, Nikolaos Maglaveras

**Affiliations:** 1 Lab of Medical Informatics, Aristotle University of Thessaloniki, Thessaloniki, Greece; 2 Sleep Unit, Pulmonary Department, General Hospital “G. Papanikolaou,” Thessaloniki, Greece; 3 Pulmonary Department, Aristotle University of Thessaloniki, Thessaloniki, Greece; 4 1st Cardiology Department, General Hospital “AHEPA,” Thessaloniki, Greece; Charité—Universitätsmedizin Berlin, GERMANY

## Abstract

**Introduction:**

Obstructive Sleep Apnea (OSA) is a common sleep disorder requiring the time/money consuming polysomnography for diagnosis. Alternative methods for initial evaluation are sought. Our aim was the prediction of Apnea-Hypopnea Index (AHI) in patients potentially suffering from OSA based on nonlinear analysis of respiratory biosignals during sleep, a method that is related to the pathophysiology of the disorder.

**Materials and Methods:**

Patients referred to a Sleep Unit (135) underwent full polysomnography. Three nonlinear indices (Largest Lyapunov Exponent, Detrended Fluctuation Analysis and Approximate Entropy) extracted from two biosignals (airflow from a nasal cannula, thoracic movement) and one linear derived from Oxygen saturation provided input to a data mining application with contemporary classification algorithms for the creation of predictive models for AHI.

**Results:**

A linear regression model presented a correlation coefficient of 0.77 in predicting AHI. With a cutoff value of AHI = 8, the sensitivity and specificity were 93% and 71.4% in discrimination between patients and normal subjects. The decision tree for the discrimination between patients and normal had sensitivity and specificity of 91% and 60%, respectively. Certain obtained nonlinear values correlated significantly with commonly accepted physiological parameters of people suffering from OSA.

**Discussion:**

We developed a predictive model for the presence/severity of OSA using a simple linear equation and additional decision trees with nonlinear features extracted from 3 respiratory recordings. The accuracy of the methodology is high and the findings provide insight to the underlying pathophysiology of the syndrome.

**Conclusions:**

Reliable predictions of OSA are possible using linear and nonlinear indices from only 3 respiratory signals during sleep. The proposed models could lead to a better study of the pathophysiology of OSA and facilitate initial evaluation/follow up of suspected patients OSA utilizing a practical low cost methodology.

**Trial Registration:**

ClinicalTrials.gov NCT01161381

## Introduction

Obstructive Sleep Apnea-Hypopnea (OSA) affects approximately 17% and 9% of middle-aged men and women. Deteriorations in patients’ health [1, 2], cognitive status [3] and quality of life [4] reveal the importance of a normal sleep pattern. Early detection is necessary for effective management [5, 6].

Although full polysomnography remains the gold standard for diagnosis, overnight oximetry [7] or level III/IV portable devices [8–10] have been utilized for alternative initial assessment of patients, as the number of those subjects is increasing. Their effectiveness has been controversial and specialized decision making algorithms are needed to ensure efficacy [6, 11]. These measurements only consider the reduction in oxygen saturation or linear features of sleep recordings, not providing insight to underlying pathophysiology. Researchers have demonstrated alternative methods for OSA screening [10, 12, 13] during sleep with variable accuracy.

Normal sleep is actively regulated modulating autonomous nervous system functions such as temperature, respiration and circulation [14]. The regulation of this activity is a nonlinear deterministic behavior [15]. As a result, researchers have tried to apply measures of nonlinear dynamics to EEG and ECG signals from polysomnography [16]. These dynamics have not been explored in pathological respiratory signals in OSA nor applied to novel detection methodologies. There are methodologies in the literature expressing Heart Rate Variability [17, 18] for the detection of apneas, indicating the validity of such techniques for OSA screening with promising results. Specialized and adequately tested data mining and algorithmic development tools are also of big importance in the context of assessing abnormalities in the observed human biosignals.

A simple linear equation predicting AHI based on nonlinear analysis would be useful as an assessment method for persons at risk of OSA, given a reasonably high accuracy. Certain efforts in this field include clinical predictive models for risk estimation for OSA [18]. However their accuracy is debatable and their equations lack nonlinear variables revealing the autonomic control status of respiration.

In this paper, we exploit a limited number of respiratory biosignals recorded during sleep to extract predictive measurements for OSA after applying nonlinear analysis on obtained data. The desired classification techniques were produced using specialized data mining and statistical tools. Our goal was to classify potential OSA patients into severity groups using a decision tree producing algorithm based on nonlinear analysis of a limited number of respiratory signals alone, instead of utilizing full polysomnography and develop a robust algorithmic solution to the problem of OSA detection using advanced nonlinear measurements and analysis techniques. The innovation of the study lies mainly in the use of novel nonlinear indices to describe the altered autonomic control during sleep disordered breathing, using only small segments of nocturnal respiratory signals.

## Patients and Methods

### Recording & pre-processing

The study included the assessment of patients with full night polysomnography and then evaluating the nonlinear characteristics described below. All patients accepted to sign the informed consent form. The study protocol was approved by the ethics committee of the hospital (Scientific Council of "G. Papanikolaou" General Hospital of Thessaloniki, Greece) in 2005. Patients referred to a tertiary hospital Sleep Unit during three years (2006–2009) were included. One out of every five consecutive patients was selected to enter the protocol. The study population was selected until the subject No 100 and their allocation to the groups of normal or OSA sufferers was based on the results of the polysomnography. The subjects were interviewed and enrolled by the first author of this paper and the manual scoring of the sleep studies was performed by the same experienced medical doctor (VT) who was blinded to whether the subjects were participating in the study or not. All subjects had symptoms consistent with OSA, i.e. reported pauses in breathing during sleep, daytime sleepiness and fatigue, frequent arousals at night and snoring at variable percentages. Dementia, neuromuscular disorders, overlap syndrome or severe cardiac problems were exclusion criteria [19]. Finally, medications that affected the sleep patterns were also exclusion criteria. The procedures related to this study were officially registered (www.clinicaltrials.gov/ct2/show/study/NCT01161381). There was small delay in the registration of the study due to the fact that the registration was not mandatory for the approval by the national ethics committee and was sought when the preliminary results for the trial were to be published. The full study protocol can be accessed as Supporting Information (S1 Text). The authors confirm that all ongoing and related trials for this intervention are registered.

The subjects underwent overnight attended polysomnography (Somnologica, Flaga; Iceland) according to standard criteria [20]. For statistically significant results, the sample size was calculated assuming a mean difference of 0.15 and a standard deviation of 0.2 for the nonlinear parameters like DFA α factor between normal subjects and patients. A sample size of 100 subjects (25 controls/75 patients) would have 90% power to detect differences (p = 0.05), therefore it was the number of participants we decided to follow.

From two respiratory signals (nasal cannula flow-F and thoracic belt movement-T), 3 nonlinear indices (Largest Lyapunov Exponent-LLE, Detrended Fluctuation Analysis-DFA and Approximate Entropy-APEN) were extracted. Oxygen saturation signal (SpO_2_) from pulse oximetry was also analyzed. The signals had a mean duration of 317.5 minutes and were exported in European Data Format to be processed by signal processing software (Matlab by Mathworks Inc.). The calculation of LLE required a command line application by Rosenstein [21] and a spreadsheet program.

Statistical analysis was performed with SPSS, Version 15.0 (SPSS Inc, Chicago, Illinois). Correlations between the studied parameters were explored with Pearson’s correlation test and differences in the mean observed values among OSA severity groups were analyzed using Student’s t-test or Mann-Whitney test according to whether the data had a normal distribution or not. Normality was tested with the Kolmogorov-Smirnoff test. The statistical significance level was set at p<0.05. The predictive model was created utilizing the linear regression tool.

### Feature extraction

**Detrended Fluctuation Analysis (DFA)** [22, 23] is a very useful tool in revealing long-range correlations in time series. Briefly, the time series to be analyzed (with N samples) is first integrated. Then, the integrated time series is divided into boxes of equal length, n. In each box, a least squares line is fit to the data (representing the trend). The y coordinate of the straight line segments is denoted by yn(k). Next, the integrated time series, y(k) is detrended, by subtracting the local trend, yn(k), in each box. The root-mean-square fluctuation of this integrated and detrended time series is calculated by

$$F(n)=\sqrt{\frac{1}{N}\sum_{k=1}^{N}{\left[y(k)-y_{n}(k)\right]}^{2}}$$

This computation is repeated over time scales to characterize the relationship between F(n), the average fluctuation, and the box size, n. Typically, F(n) will increase with box size. A linear relationship on a log-log plot indicates power law (fractal) scaling. Under such conditions, the fluctuations are characterized by a scaling exponent, the slope of the line relating log F(n) to log n.

The “time boxes” for signal analyses with DFA were selected at 8, 30, and 100 seconds, representing mean durations of 2, 6 and 24 breaths respectively. Twenty minutes long time series were used for the calculation of DFA from F and 240 minutes from T signals. Τhe two types of signals differ in length due to the large size of the flow signals (200Hz sampling). The measurements produced from each signal were the *DFAfast* value, representing the power law slope on the medium to fast time scales, and *DFAslow*, showing the slope on the slow to medium time scales. Apart from these measurements, additional derived parameters were introduced: dDFA_f, representing the difference between DFAfast and DFAslow value from F and mDFA_f, produced from the mean value of the two previous parameters. Similar parameters were derived from the thoracic DFA measurements (dDFA_t, mDFA_t). Also, dDFA_f2 and mDFA_f2 refer to the analogous parameters when the F signals were reduced to one fourth of their initial duration (achieved by keeping every fourth value of each time series from F), thus allowing for signal analysis of 80 minutes in each case. Finally dmDFA_t2 and mmDFA_t2 were introduced by taking the difference between or the mean value of mDFA_f2 and mDFA_t [19].

In dynamical systems, entropy is the rate of information production. Methods for estimation of the entropy of a system represented by a time series are not well suited to analysis of the short and noisy data sets encountered in biological studies. **Approximate Entropy (ApEn)** [24, 25], introduced by Pincus, is a measure of system complexity closely related to entropy, easily applied to clinical time series.

The method examines time series for similar epochs: more frequent / similar epochs lead to lower values of ApEn. Given *N* points, the family of statistics ApEn(*m*, *r*, *N*) is approximately equal to the negative average natural logarithm of the conditional probability that two sequences that are similar for *m* points remain similar, within a tolerance *r*, at the next point. Thus a low value of ApEn reflects a high degree of regularity. For this method, the measurement parameters selected were: m = 2, r = 0.2, N = total sleep recording, values coinciding with previously used ones [14]. Alternative values showed no significant alteration in results. Again, additional related parameters were derived from the original APEN_high and APEN_low values from every signal: dAPEN and mAPEN.

**Largest Lyapunov exponent (LLE)** is a quantitative measure of sensitive dependence on the initial conditions for a dynamic system. An increase in LLE is indicative of more complex behaviour [23]. A robust and practical method for reliably calculating LLE was proposed by Rosenstein *et al*. For its estimation, consider two points in a space: X_0_ & X_0_ + Dx_0_, each of which will generate an orbit in that space using some equation or system of equations. These orbits can be thought of as parametric functions of a variable like time. Using one of the orbits as a reference orbit, the separation between the two orbits is also a function of time. Because sensitive dependence can arise only in some portions of a system, this separation is also a function of the location of the initial value and has the form Dx(X_0_, t). In a system with attracting fixed points or attracting periodic points, Dx(X_0_, t) diminishes asymptotically with time. If a system is unstable, the orbits diverge exponentially for a while, but eventually settle down. For chaotic points, the function Dx(X_0_, t) will behave erratically. It is thus useful to study the mean exponential rate of divergence of two initially close orbits using the formula:

$$\lambda =\underset{\begin{array}{c} t\to \infty \\ |{\mathrm{\Delta x}}_{0}|\to 0 \end{array}}{\lim}\frac{1}{t}\;ln\frac{\left|\Delta x({Χ}_{0},t)\right|}{\left|{\Delta x}_{0}\right|}$$

This number, called the Lyapunov exponent "λ", is useful for distinguishing among various types of orbits. For LLE measurements [26], on F signals, the method was applied for periods of 20 minutes (LLEf), whereas 170-minute periods were used for T signals (LLEt); dLLE, mLLE, LLEf2, dLLE2 and mLLE2 represent additional parameters derived from the above.

T90 (Time with SpO2<90% as a percentage of total studied time) was used as a linear feature aiding the production of the predictive models. It depicted the degree of hypoxemia during sleep.

The same time windows were selected from all the analyzed biosignals, starting 1 hour after initiation of the sleep study.

In this analysis, we have chosen the *classifier subset evaluator* as a feature evaluator and *BestFirst* as a search method. The *classifier subset evaluator* evaluates attribute subsets on training data or a separate hold out testing set. Furthermore, we have used the J48 classifier as a classifier to estimate the 'merit' of a set of attributes. The *BestFirst* searches the space of feature subsets by greedy hill-climbing augmented with a backtracking facility.

### Classification techniques

C4.5 Decision tree learning is a method of inductive inference, robust to noisy data and capable of learning disjunctive expressions [27]. It is also a method for approximating discrete-valued functions, in which the learned function is represented by a decision tree. Learned trees can also be represented as sets of if–then rules to improve human readability. It constitutes a type of heuristic search providing decision trees, applied to a wide range of medical diagnostic tasks [27–29].

The performance of each classifier was assessed with a stratified 10-fold cross-validation method. This approach has the advantage that all the data is used for model evaluation, instead of simply splitting the data into testing and training sets. The data was divided into 10 equal sized fragments, each of which was in turn used as an independent test set, while the other fractions were used for training the classifier. Classification error was estimated as the average performance over the 10 test sets. This allows the building of a predictive model without the need of using a separate training set of patients, whilst its efficacy remains high in real conditions [30]. Classification and feature selection were performed with the free software Weka 3.5.8 (University of Waikato, New Zealand) [31].

## Results

**Fig 1:**
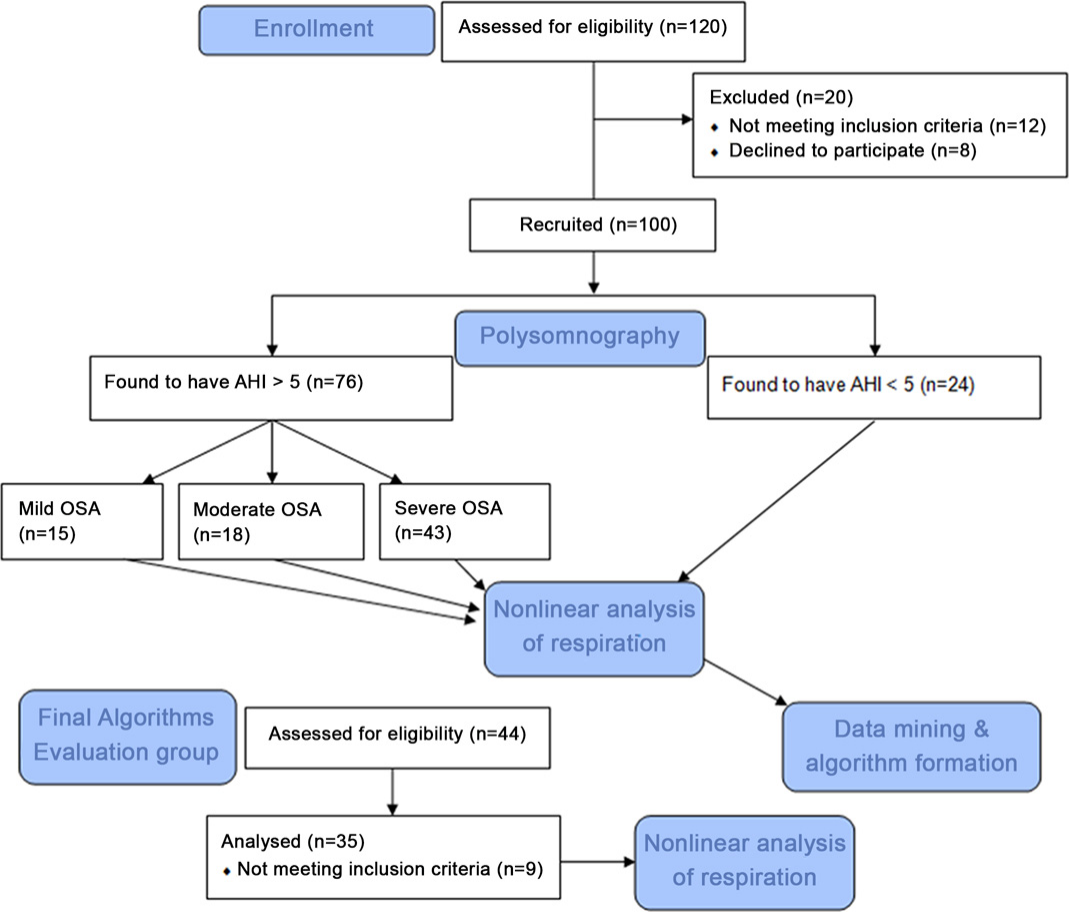
The Flow Diagram of the study.

One hundred subjects were initially included in the study. In total, 24 subjects were found to be normal (AHI<5/hour) and 76 suffered from OSA after manual scoring of the full polysomnographic recordings. The study flow diagram is provided in Fig 1. Table 1 summarizes the descriptive statistics from the initial pool of 100 patients.

**Table 1:** Descriptive statistics from the study population (N = 100). BMI = Body Mass Index, T90 = Time with SaO2<90% (in percentage of Total Sleep Time), AHI = Apnea-Hypopnea Index (in events/hour), AI = Apnea Index, HI = Hypopnea Index, LLE = Largest Lyapunov Exponent, f = flow signal, t = thoracic belt signal, DFA = Detrended Fluctuation Analysis α factor (slow-fast), APEN = Approximate Entropy (see text & Supporting Information for further details).

	Minimum	Maximum	Mean	Standard Deviation
AGE	16.0	83.0	48.4	13.6
EPWORTH	0.0	22.0	7.8	5.1
BMI	16.97	53.10	31.94	6.89
T90	0.0	100.0	23.9	31.0
AHI	0.0	135.9	33.9	31.7
AI	0.0	124.9	22.7	28.9
HI	0.0	47.3	11.2	10.6
LLEf	-0.14	3.34	0.57	0.54
LLEt	-0.08	4.45	1.03	0.71
dLLE	-2.89	1.84	-0.47	0.69
mLLE	0.20	3.10	0.80	0.52
LLEf2	0.00	1.84	0.67	0.37
dLLE2	-3.18	1.84	-0.33	0.78
mLLE2	0.26	2.86	0.85	0.41
DFA slow_f	0.01	1.64	0.24	0.22
DFA fast_f	0.02	1.46	0.28	0.29
dDFA_f	-1.17	0.44	-0.04	0.24
mDFA_f	0.01	1.48	0.26	0.23
DFA slow_f2	0.01	1.70	0.36	0.26
DFA fast_f2	0.08	1.12	0.34	0.22
dDFA_f2	-0.61	0.99	0.02	0.25
mDFA_f2	0.05	1.21	0.35	0.21
DFA slow_t	0.01	1.10	0.17	0.21
DFA fast_t	0.37	1.19	0.56	0.20
dDFA_t	-0.66	0.99	-0.33	0.29
mDFA_t	0.20	1.15	0.36	0.18
APEN low_f	-52.50	-3.21	-8.08	8.75
APEN high_f	3.22	53.63	8.40	8.31
dAPEN_f	-17.50	7.80	0.32	3.12
mAPEN_f	-8.75	3.90	0.16	1.56
APEN low_t	-209.52	-9.09	-27.76	29.68
APEN high_t	11.42	190.96	33.90	28.68
dAPEN_t	-126.15	75.04	6.14	20.19
mAPEN_t	-63.08	37.52	3.07	10.09
dmDFAt2	-0.93	0.97	-0.37	0.24
mmDFAt2	0.28	0.99	0.44	0.13

The Total Sleep Time averaged 317.6±55 minutes with a mean sleep efficiency of 91%. Of the study population, 74 were male and 26 female. The mean age and AHI were 48.4±13.6 and 33.9±31.7 respectively. Fifteen subjects had mild OSA, 18 had a moderate syndrome profile and 43 suffered from severe OSA. Only 4 participants were found to have PLMS during the sleep studies and the number was considered too small to affect the obtained results.

The Kolmogorov-Smirnoff test revealed that the variables AGE, Epworth Score, LLE derivatives (except LLEt), and most of the DFA parameters (except DFA fast_f2, mmDFAt2 and DFAfast_f) had a normal distribution.

Using the Student’s t-test between normal subjects and OSA patients, significant differences were found in age, Epworth score, dLLE, DFA fast_f2, and mDFAf2 (Table 2).

**Table 2:** t-test for Equality of Means between normal and OSA patients. Only statistically significant differences are displayed.

	t	Significance (2-tailed)	Mean Difference	95% Confidence Interval	
				Upper	Lower
**AGE**	-2.75	0.007	-8.45	-14.54	-2.35
**EPWORTH**	-4.13	0	-4.59	-6.79	-2.38
**dLLE**	-1.99	0.049	-0.32	-0.63	0
**dDFA_f**_**2**_	2.43	0.017	0.14	0.03	0.25
**mDFA_f**_**2**_	-2.29	0.024	-0.11	-0.2	-0.01

For variables that did not have a normal distribution the Mann-Whitney nonparametric test was executed, revealing the statistically significant differences in BMI, T90, LLEt, DFA fast_f, DFA fast_f_2_ and mmDFA_t2, as displayed in Table 3.

**Table 3:** Mann-Whitney nonparametric test for Equality of Means between normal and OSA patients. Only statistically significant differences are displayed.

	Mann-Whitney U	Z	Asymp. Sig. (2-tailed)
**T90**	312	-4,9	0,000
**BMI**	545	-3,0	0,003
**DFA fast_f**_**2**_	392	-4,2	0,000
**mmDFAt**_**2**_	591	-2,5	0,012
**LLEt**	622	-2,3	0,019
**DFA fast_f**	643	-2,2	0,030

The Pearson’s correlation test revealed statistically significant correlations between AHI and the parameters: Epworth (r = 0.47, p<0.01), BMI (r = 0.58, p<0.01), T90 (r = 0.7, p<0.01), dLLE (r = 0.3, p<0.01), DFAfast_f2 (r = 0.4, p<0.01), dDFA_f2 (r = -0.36, p<0.01), mDFA_f2 (r = 0.21, p<0.05), dmDFA_t2 (r = 0.34, p<0.01) and mmDFA_t2 (r = 0.51, p<0.01). Similar correlations were found for the measured Apnea Index (AI) but not for Hypopnea Index (HI).

Using linear and nonlinear data from the respiratory signals and supplying them to the data mining software, two decision trees were produced. The first presents an algorithm discriminating between normal subjects and OSA patients, aiding the decision whether to further examine a patient for OSA, constituting an initial assessment tool. On the other hand, the decision of applying certain treatment modalities depends–among others- on the severity level of the syndrome, with the patients with moderate and severe OSA (as defined by the AHI measurement) requiring initialization of more aggressive treatment, at least in most of the cases. The OSA severity is classified as follows: 5<AHI≤15 = *mild*, 15<AHI≤30 = *moderate* and AHI>30 = *severe*. The current practice is to consider CPAP or other interventions as the optimal therapy for moderate/severe OSA and conservative measures for milder forms, apart from selected cases that could benefit from alternative treatments [32]. In view of this, a second algorithm was sought to categorize patients into two groups of disease severity: normal/mild syndrome and moderate/severe OSA (calling it the CPAP treatment group).

**Fig 2:**
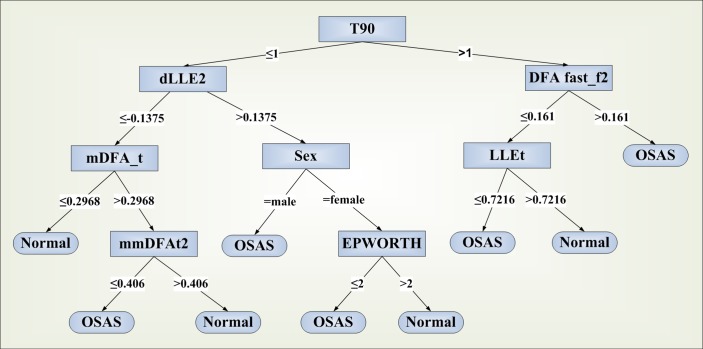
Decision tree produced by C4.5 algorithm for the classification of subjects into OSA patients or normal.

The discrimination between normal subjects and OSA patients presented a sensitivity of 74.8% and a specificity of 74% using the variables sex, Epworth score, DFA from nasal cannula flow (F) and Thoracic movement (T), LLE from both F and T and Time with SpO_2_<90% (T90) (Fig 2). The area under the ROC curve for this method was estimated to be 0.684.

**Fig 3:**
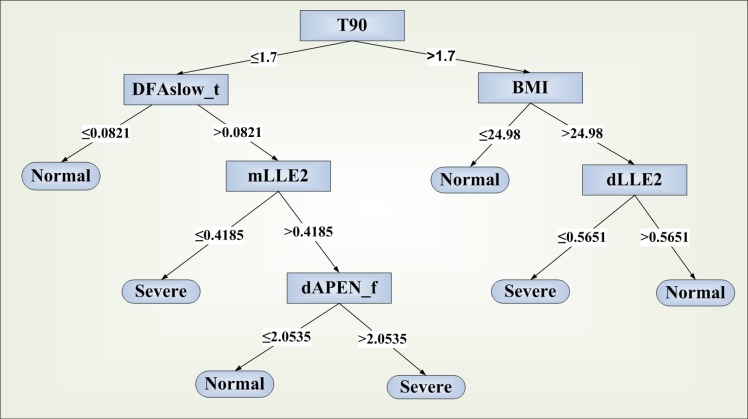
Decision tree produced by C4.5 algorithm for the classification of OSA patients into severity groups according to the need for CPAP. “Normal”: AHI < 15, “severe”: AHI ≥ 15.

The second algorithm is suitable for the classification of OSA patients into groups according to the severity of the disease (i.e. whether the AHI is below or over 15). Fig 3 shows the produced decision tree for the classification.

The classification of patients into severity groups had a sensitivity of 85% and a specificity of 85% using the variables BMI, APEN from F, LLE from both F and T, DFA from T and T90 (Table 4). The algorithm was more precise in cases with severe OSA, which would most probably need the use of CPAP devices for therapeutic purposes. The area under the ROC curve reached the level of 0.86. All the described decision tools were tested using only one or two out of the three initially selected respiratory biosignals. In all of the possible combinations the performance indicators were significantly lower than those presented above, leading to the conclusion that the selected signals are essential for a more precise discrimination.

**Table 4:** C4.5 Statistics for severity of OSA. TP = True Positive, FP = False Positive.

	**Overall Algorithm Accuracy**					
**Correctly Classified Instances %**		**Incorrectly Classified Instances %**			**Kappa statistic**	**Total Number of Instances**
85		15			0.6773	100
	**Detailed Accuracy By Class**					
**TP Rate**	**FP Rate**	**Precision**	**Recall**	**F-Measure**	**ROC Area**	**Class**
0.918	0.256	0.848	0.918	0.882	0.859	moderate/severe
0.744	0.082	0.853	0.744	0.795	0.859	normal/ mild
**0.85**	**0.188**	**0.85**	**0.85**	**0.848**	**0.859**	**Weighted Avg.**

By applying a linear regression analysis to the obtained data, the following equation was produced:

AHI=1.2*BMI+0.4*T90−18.6*dDFAf2+7.8*dLLE+60.5*mmDFAt2−27

This equation showed a sensitivity of 82% with a specificity of 57% for the cutoff value of AHI = 5/h, whether the same measures were 68% and 70%, respectively when the threshold was set at 8.

**Fig 4:**
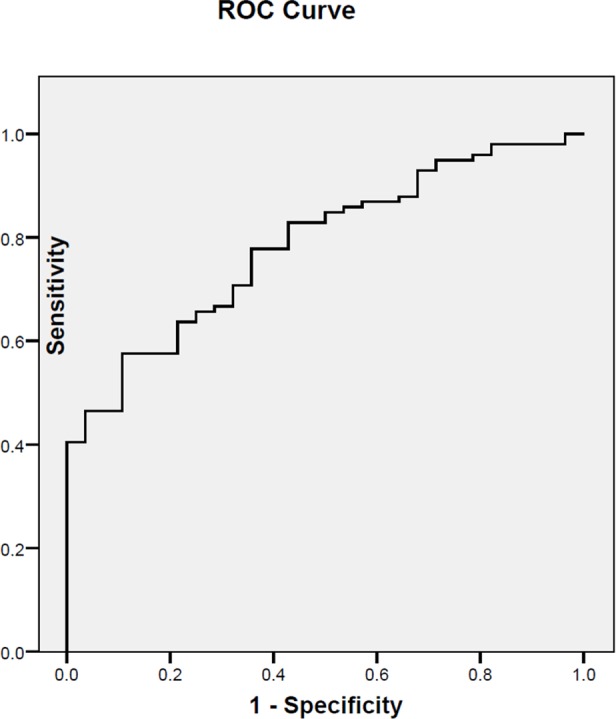
ROC Curve of the linear regression equation proposed as a prediction model for AHI.

Fig 4 illustrates the Receiver Operating Characteristic (ROC) Curve of the proposed predictive model for different thresholds of AHI. The Area Under the Curve was 0.77.

After the initial development and evaluation of our methodology, the predicting algorithms were tested against standard polysomnography in a separate group of 35 consecutive patients referred to the same clinic and with the same inclusion/exclusion criteria. This pool of patients had a mean age of 46.46 years, with a BMI of 32.55. The male/female ratio was 27/8. The sleep study performed showed an average AI of 34.026 episodes/hour with seven of them being normal and 28 having a measured AHI>15/hour. Of the latter, five suffered from mild OSA, five from moderate and the remaining 18 from severe. The decision tree discriminating OSA patients from normal showed a sensitivity of 90% and a specificity of 60%, with an overall diagnostic accuracy of 85.7%. The second algorithm used to classify patients into groups of severity showed sensitivity and specificity levels of 92.3% and 88.9%, respectively and a diagnostic accuracy of 91.4%.

**Fig 5:**
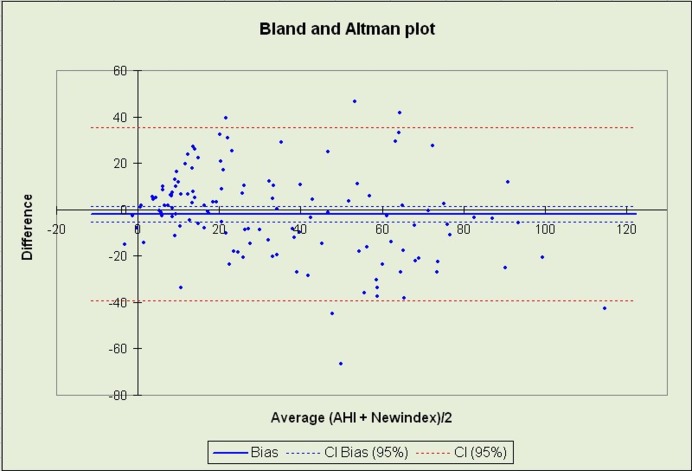
Bland & Altman Plot for the detection of OSA with the proposed linear equation versus standard overnight polysomnography.

The linear equation for the estimation of AHI produced results that correlated well with those obtained by polysomnography (correlation coefficient = 0.815, p<0.001). Concordance with the AHI values extracted by manual scoring of polysomnographic studies was controlled using a Bland and Altman plot [33] as seen in Fig 5. When the calculated AHI threshold was set to 8 for the diagnosis of OSA, the obtained diagnostic accuracy was 88.6%, with sensitivity and specificity levels of 92.9% and 71.4%, respectively.

An interesting observation was the approach of certain nonlinear values to the previously published values for the loop gain of the respiratory system (an important variable describing the respiratory system balance point) in severe or mild OSA patients [34]: In severe patients, mean LLEf was 0.62 and mmDFAt2 was 0.52, values close to 0.6 reported for the loop gain of this group. On the other hand, the respective values in mild sufferers and normal subjects were 0.4 and 0.39, very close or equal to the reported value of 0.4 for the loop gain in the mild OSA group of patients.

## Discussion

We have developed and tested a predictive model for the presence and severity of OSA using a simple linear equation, with two additional decision trees that could manage patients undergoing sleep studies limited to 3 respiratory recordings with the help of nonlinear features extracted from the aforementioned biosignals. The innovation of our findings lies in the fields of knowledge extraction techniques as well as the exploitation of nonlinear indices which approach the underlying pathophysiology more effectively than conventional measurements. The selected biosignals indicate disturbed respiration during sleep reliably and are considered practical to use. Utilization of these signals alone for accurate detection of sleep apneas/hypopneas can be used instead of the expensive and time consuming full polysomnography or other methods requiring more signals [9, 11, 35]. Other studies utilizing nonlinear features of sleep recordings [17, 18] have also shown positive findings but had never exploited respiratory signals and their accuracy levels were lower (accuracy 78–80%) [17] or equal to ours (decision tree diagnostic accuracy 91.2%) [18].

This study adds to the efforts to develop sets of algorithmic solutions to the issue of categorizing the severity of OSA in sleeping subjects. The described algorithms and equations could improve the accuracy of portable monitors for sleep apnea in automated detection of the syndrome and this could be achieved by recording even small time windows (in contrast to the current practice of full night recordings), as shown in our methodology. Previously published efforts [18] have used full night recordings without superior results as concerns the prognostic value. Modern portable devices able to measure the studied parameters are already under development or have already reached the market [10] and are bound to bring a new era of remote signal acquisition in home care. The integration of the described algorithms in such devices could facilitate screening for OSA in remote areas, without the need for specialized sleep units, enhancing telemedicine capabilities.

The proposed algorithms could be used for automated evaluation of patients suspected to have OSA. The results show promising levels of accuracy in this field and the equipment needed for this detection is generally of low cost and easy to operate. Studies with alternative screening/detection techniques like overnight oximetry have shown lower sensitivity (approximately 60%-88% at the threshold of AHI = 5) [7, 9, 12, 14–17, 36] when compared to the proposed linear prediction model, while the proposed decision tree discriminating between patients in need of CPAP or not has demonstrated superb precision and recall features [14–16]. To our knowledge, there has not been evaluated any similar model for therapeutic decision making without the utilization of full polysomnography.

Other researchers have performed measurements of nonlinear indices of human respiration during sleep [14–16]. We have found weaker long term correlations in the nasal flow signals [14] using the DFA technique, but these were significantly enhanced in OSA patients, allowing us to exploit these differences in developing a classification tool. This increase in long term correlations of respiration (depicted by higher DFA or dDFA values) is indicative of impaired long term regulation from the autonomous nervous system, i.e. a more constrained autonomic influenced control of breathing due to increased influence of cortical stimuli as a result of micro arousals at the apneic events. This complies with indications of increased regularity and loss of normal variability during deep sleep (shown by the lower APEN values in respiratory signals), as a result from reduced respiratory motor output concerning phrenic and hypoglossal activity. Moreover, increased dependence on initial conditioning was found in the patients’ system (depicted in higher LLE values). This finding could be an indicator of dynamic imbalance between ventilatory pump drive and upper airway muscle activity, in agreement with higher loop gains in the system of severe OSA sufferers found by other researchers [34]. The correlation between the derived values of LLEf, mmDFAt2 and the loop gain of OSA patients [34] is suggestive of a satisfactory approach of the underlying pathophysiology by our models. The observed increased sympathetic excitation in these patients even at wakefulness [37] due to intermittent hypoxia and the resulting oxidative stress may also cause an impaired initial conditioning of the respiratory system, precipitating pharyngeal collapse, reflected in the higher LLE values at OSA patients. Finally the use of BMI in the proposed predictive models reflects the anatomical aspect of the upper airway collapsibility that plays a role in the presence of the syndrome. We have not found any published references to DFA measurements for thoracic belt signals or APEN estimations from either of the respiratory recordings.

Of special interest is the fact that the nonlinear indices proved to be useful in classifying patients into severity groups regardless of their sleep staging. They allowed for conclusions about OSA severity without the simultaneous analysis of heart rate variability or any neuromuscular recording. The studied parameters seem to account for a generalized description of the intrinsic autonomic control of breathing during sleep affected by the disordered sleep pattern of OSA, demonstrating significant changes when the syndrome is present. This could open an issue of new types of research in respiratory physiology during sleep disorders, as nonlinear analysis is potentially more suitable for describing the dynamic behavior of human respiration in abnormal conditions, as shown recently by various researchers.[28]The complex cardiorespiratory dynamics exhibit dependence on multiple intrinsic oscillators and the nonlinear traits are considered better descriptors of the autonomic nervous effects on them. The nonlinear features can play an important role in exploring respiratory system nonstationarities emerging from transition between sleep stages and responses to internal or environmental influences. Furthermore, the breath to breath variability (as described by some of the proposed indices) can explain either central neural mechanisms or the instability in the chemical feedback loops, for example in central or obstructive sleep apnea. The expansion of such observations can help improve virtual respiratory system modelling efforts which are based on nonlinear lung compliance and airway resistance. These models have already been used in studying the effect of CPAP devices on obstructed airways.[38]

Among the weaknesses of this work is the absence of measurements in an uncontrolled setting like the home of the subjects, which could cause lower quality of obtained biosignals compared to what is recorder in a sleep lab. The nonlinear indices have been shown to be more immune to signal noise than conventional measurements, a fact that could compensate for this difference. The issue is going to be further examined in future studies in multiple settings. The relatively low computational power of the personal computers used, in conjunction with the increased computational capability that some of the above methods (like DFA) require is also a limitation. We were able to study fixed time windows of limited duration, while more prolonged analyses would be desirable. On the other hand, the applied time windows proved to be adequate in discrimination between normal or patients, although the effect of longer windows on the accuracy of the prediction is an open issue.

We have chosen to consider the results of the studied population and not those from the excluded patients (who actually suffered from OSA at a percentage of 50%) because the goal was to correlate the performance of the algorithms and the nonlinear traits to the physiology of specific disease states and thus we had to exclude as many confounding factors as possible. Further multicentric analyses could show the effectiveness and reproducibility of the proposed method and also explore the trends in the accuracy in groups of patients with other comorbidities (e.g. congestive heart failure, overlap syndrome).

## Supporting Information

S1 Text The protocol of the study. (DOCX)

S1 Table The CONSORT Checklist of the study. (DOC)

S2 Table The data set from the study. (XLS)
