# Supplementary material for: Evaluation of a Decision Support System for Obstructive Sleep Apnea with Nonlinear Analysis of Respiratory Signals
Source: PLoS One. 2016 Mar 3;11(3):e0150163. doi: 10.1371/journal.pone.0150163 (PMC4777493; doi:10.1371/journal.pone.0150163)
Supplement: S1 Text — (DOCX) [file pone.0150163.s001.docx]

### Aim

The aim of this prospective study is the analysis of the respiratory biosignals that will be obtained during polysomnographic acquisitions on patients with Obstructive Sleep Apnea Hypopnea Syndrome (OSAHS) and healthy people with the use of signal processing techniques based on complexity theory. The extrapolated measurements from persons suffering from OSAHS will then be compared to those from healthy volunteers, in order to enable development of methodologies capable of detection of the disease using only the respiratory recordings. Moreover the nonlinear characteristics of the signals will be linked to the pathophysiology of the obstruction of the upper airway and its modelling.

### Sleep Unit

The patients to be studied will be recruited at the outpatient clinic of the Sleep Unit of the 2^nd^ Pulmonary Department of “G. Papanikolaou” General Hospital of Thessaloniki, Greece. These patients must present with symptoms consistent with OSAHS. They may either visit the sleep unit on their own or be referred to it by other sleep specialists/ doctors from other specialties. The patients will be residents of the wider area of Thessaloniki and central Macedonia. For randomization purposes, every fifth patient referred to the sleep lab will be asked to participate in the study. Each potential participant will be fully briefed about the details of the study and if accepted, he/she will be expected to fill the consent form with the presence of a study investigator. The full consent form can be found in Supplement A.

### Inclusion – Exclusion Criteria

The ***inclusion criteria*** for the study are the following:

1. Voluntary visit to the outpatient clinic of the sleep unit of the 2^nd^ pulmonary clinic
2. Subjective symptoms consistent with OSAHS
3. Age over 16 years old
4. First visit to a sleep laboratory

The ***exclusion criteria*** are:

1. Presence of dementia of any level
2. Serious anatomy disorder at the region of head and neck
3. Presence of neuromuscular disorders
4. History of obesity in conjunction with hypoventilation
5. Presence of overlap syndrome (combination of COPD and SOAHS)
6. Severe cardiologic disorder
7. History of CPAP/ BiPAP for any reason
8. Use of sleeping pills or other medication targeting the central nervous system during the last month
9. Failure of patient to comprehend or provide consent to the specific study

### Patients – Methods

### Study characteristics

The clinical trial will be a prospective randomized controlled trial with a control group and will be held at the Sleep Laboratory of the 2^nd^ Pulmonary Department of “G. Papanikolaou” General Hospital of Thessaloniki, Greece beginning in the year 2005.

For every patient who reaches the sleep laboratory and will be randomly allocated to the study the following control measurements will be executed:

1. Comprehensive clinical history with regard to symptoms related to sleep, potential comorbid situations and use of medication
2. Clinical examination and measurement of somatometric characteristics
3. Filling of Epworth daytime sleepiness scale (properly translated and validated to Greek)
4. In case of suspicion for COPD presence, measurement of arterial blood gases and spirometry testing
5. Explanation of the study protocol and signing of the informed consent form

### Recording and pre-processing

In order for statistically significant results to be exploitable, the study sample size was calculated, assuming a mean difference of 0.15 with a standard deviation of approximately 0.2 between healthy and OSAHS patients for nonlinear parameters like the factor DFA α. A sample size of at least one hundred participants (allocated 25 in the control group and 75 in the intervention - patients group) will yield under these conditions a statistical power of approximately 90% for the detection of differences at the lever of statistical significance set at 5%. Therefore, the target study population will be 100 recruited persons in the aforementioned allocation distribution.

All participants will have to present with symptoms consistent with obstructive sleep disorders and not have any significant comorbidities. In case a subjects has any of the exclusion criteria present, he/she will be excluded from the study and the next examined person in the outpatient clinic of the sleep unit will be asked to participate in the trial. The subjects will undergo full polysomnography which will be attended in the collaborating sleep unit for the whole night without use of possible split night studies. The examination will be using equipment from Somnologica (7000, Flaga; Iceland) according to standard analysis criteria and will include respiratory recordings from the thoracic and abdominal wall movement, airflow from the nostrils using a nasal pressure cannula, as well as pulse oximetry. The apnea and hypopnea episodes will be defined according to internationally established scoring criteria. All the sleep recordings will be manually scored by the same experienced sleep medicine doctor.

Three nonlinear indices (Largest Lyapunov Exponent-LLE, Detrended Fluctuation Analysis-DFA and Approximate Entropy-APEN) will be extracted from two of the respiratory signals from polysomnography (the airflow from the nasal cannula flow-F and the thoracic wall movement with the help of the thoracic belt – thoracic belt movement-T). Also, the Hemoglobin Oxygen Saturation signal-SpO_2_) from pulse oximetry (finger probe) will be used. The above signals will initially be exported from the sleep analysis software in European Data Format (EDF) in order to be further analyzed using signal processing software solutions like Matlab by Mathworks Inc. in personal computers. The calculation of LLE requires the use of an application that operates in command line (MS-DOS type) plus a spreadsheet software like Microsoft Excel.

The Apnea – Hypopnea Index-AHI will be used as the primary measure of OSAHS severity, since in the present study full polysomnography recordings will be utilized and manual scoring of the signals will be the scoring method.

### Feature extraction

As concerns the analysis methods and the parameters selection for their setting, the following information is explanatory:

For the method of Detrended Fluctuation Analysis (DFA): The “time boxes” for the signal analyses in our study will be selected at time periods of 8, 30, and 100 seconds, which represented the mean duration of 2, 6 and 24 breaths respectively. Twenty minutes long time series will be used for the calculation of DFA from Flow signals (F) whereas the same duration for the Thoracic belt signals (T) will be approximately 240 minutes. The two types of signals differ in length due to the limited computational capabilities of the personal computers used, because of the large size of the flow signals (resulting from the 200Hz sampling in this case). The DFA measurements which will be produced from each signal are the DFA fast value, representing the power law slope on the medium to fast time scales, as well as DFA slow, showing the slope on the slow to medium time scales. Apart from these initial measurements, additional derived parameters will be introduced: dDFA_f, which represents the difference between DFA fast and DFA slow value from F and mDFA_f, which is produced from the mean value of the two previous parameters. Similar parameters derive from the thoracic DFA measurements (dDFA_t, mDFA_t). Also, dDFA_f2 and mDFA_f2 refer to the analogous derived parameters when the F signals are reduced to one fourth of their initial duration (this was achieved by keeping every fourth value of each time series from F), thus allowing for signal analysis of 80 minutes in each case. Finally dmDFA_t2 and mmDFA_t2 are introduced by taking the difference between or the mean value of mDFA_f2 and mDFA_t, respectively.

The values selected in our study concerning the analysis of the Approximate Entropy (ApEn) are: m=2, r=0.2, N=the total sleep recording. These values coincide with previously used ones, whilst alternative values showed no significant alteration in the resulting APEN figures in preliminary recordings. Again, additional related parameters will be derived from the original APEN_high and APEN_low values from every signal: dAPEN or mAPEN reflecting the differences or the means of the analogous APEN values.

For the variable Largest Lyapunov exponent-LLE, a robust and practical method for reliably calculating LLE was proposed by Rosenstein *et al.* For the flow signals, the method will be applied for periods of 20 minutes (*LLEf*), whereas 170-minute periods will be analyzed for the thoracic belt signals (*LLEt*). The additionally derived measures are the following: *dLLE* equals LLEf minus LLEt, whilst *mLLE* is the average of these two measurements. Similarly, *LLEf_2_, dLLE_2_* και *mLLE_2_* represent the respective measurements when the shortened Flow signals lasting 80 minutes are used (using a sampling rate equal to ¼ of the original).

The value *T90* (Time with oxygen saturation - SpO_2_<90% as a percentage of total recorded time) will also be used as a linear trait aiding the creation of predictive models, as it is closely related to clinical presentation of airflow limitation and the resulting hypoxemic events in patients.

The same time intervals will be selected for the analysis of all the aforementioned parameters, i.e. time windows commencing 1 hour after the onset of the recordings. This way the selected signals will reflect a time period during which the subjects are expected to have fallen asleep and also to have completed a full sleep cycle.

### Statistical analysis

The basic statistical analysis will be conducted with the statistical package SPSS for Windows, version 15.0 (SPSS Inc., Chicago, Illinois). Correlations among various measured or extracted parameters will be evaluated with the Pearson’s correlation test, while possible differences in the mean values of measurements among different subgroups of OSAHS patients or healthy subjects will be assessed with the Student’s t-test for continuous variables with normal distribution, or with the Mann-Whitney’s test for variables without a normal distribution. When the comparisons are made for more than two subgroups, the respective statistical tests will be One Way ANOVA or Kruscal-Wallis ANOVA. The test for normal distribution of the examined variables will be one sample Kolmogorov-Smirnov’s test. The level of statistical significance will be set at the level of p<0.05. Potential linear predictive models for the severity of OSAHS will be based on the linear regression tool.

The mean values of all examined parameters will be calculated for all the patients suffering from OSAHS and these mean values will be compared to the same values calculated for the healthy volunteers. For the parameters that will have statistically significant differences between the two groups, further analysis will take place with the use of specialized data mining applications. The goal will be to draw complex medical decision trees which will aid the classification of subjects in OSAHS severity groups or the creation of linear regression tools capable of calculating the Apnea-Hypopnea Index.

# Supplement Α

#

# INFORMED CONSENT FORM

FOR PARTICIPATION IN A CLINICAL STUDY

**1.  GENERAL INFORMATION**

*Please read the following information about the study you were invited to participate carefully:*

**1.1** This study is conducted as a part of a PhD thesis by Dr Evangelos Kaimakamis, who is a Medical Doctor and holder of a Master’s Degree in Medical Informatics from the medical School of Aristotle University of Thessaloniki, Greece.

**1.2** The study protocol has been approved by the ethics committee of “G. Papanikolaou” General Hospital of Thessaloniki.

**1.3** The research will take place in the Sleep Unit of the 2^nd^ Pulmonary Department of “G. Papanikolaou” General Hospital. The data processing and analysis of results will be realized in the Lab of Medical Informatics of Aristotle University of Thessaloniki.

**1.4** The responsible person for the conduction of the study, the data collection and the processing of medical data is Dr Evangelos Kaimakamis.

**2.  DETAILED INFORMATION**

*Detailed information on the clinical trial follow:*

**2.1 Study title:**

Study of the respiratory signals of patients with Obstructive Sleep Apnea-Hypopnea Syndrome using Complexity theory.

**2.2 What is the aim of the study?**

This study aims to discover whether the respiratory signal (the recording of human breathing) is altered in patients with Obstructive Sleep Apnea-Hypopnea Syndrome (OSAHS) compared to the signals from healthy volunteers, as concerns its nonlinear characteristics. This will be evident after appropriate signal analysis of respiration with the use of specialized methodologies based on Complexity Theory.

The ultimate target is the development of novel methodologies for easier diagnosis and evaluation of the progress of OSAHS.

**2.3 Who can participate in the study?**

In this study every person who is being examined at the sleep unit of the 2^nd^ Pulmonary Department of “G. Papanikolaou” General Hospital is eligible to participate. The subjects must be suspected to suffer from OSAHS without restrictions in gender or age. Certain more detailed inclusion/exclusion criteria apply.

**2.4 Why should I participate?**

Your participation will help us to better collect biomedical data that are crucial for the drawing of medical conclusions about the alteration of human respiration in this specific disease. This way it will be possible to develop novel tools for the diagnosis and the evaluation of the syndrome.

By taking part in the study you will not be asked to do anything more than what you would do anyway, whereas your personal data will be secured as fully confidential.

**2.5 Am I obliged to participate? If I agree to participate and change my mind afterwards, can I withdraw?**

You have absolutely no obligation to participate in this study. It is purely a personal choice of yours and you have the right to refuse. In case you agree to participate but you change your mind later, you retain your right to withdraw at any time and demand the deletion of your medical data from the study database.

**2.6 What exactly should I do and what procedures will be applied to me if I accept to participate in the study?**

You will not be asked to do anything more than undergo a full night polysomnography in the sleep lab of the 2^nd^ Pulmonary Department of “G. Papanikolaou” General Hospital. During this examination, we will be recording, among others, your respiration signal, which will be later extracted to be further analyzed. This signal will be processed with the use of special software in computers and will be compared to analogous signals obtained by healthy volunteers. The collected data will be correlated with age, sex, health status etc. And proper scientific outcomes will be extracted.

**2.7 Will I face any kind of danger during the study?**

No, the participation in this study does not pose any threat to your health.

**2.8 How will the privacy of my medical data be secured?**

This medical study is conducted by a certified medical doctor who is bound with the obligation to secure the privacy of medical data and who is cooperating with the University of Thessaloniki for the realization of this study. Moreover, the latter has been approved for privacy issues by the ethics committee of 2^nd^ Pulmonary Department of “G. Papanikolaou” General Hospital. Finally, the medical researchers are interested in the biosignals processing and not the personal data of the participants.

**2.9 What kinds of benefits will I have by participating in this study?**

Your participation will have no impact whatsoever on the examination process by the sleep unit and no further benefits are expected for your participation in the study.

**2.10 What kinds of benefits will other people have from this study?**

The expected outcomes of this medical study will potentially help many people who suffer from OSAHS through the alternative way of diagnosis and evaluation of the syndrome.

**2.11 Will I be paid for my participation?**

No, the participation in such a medical study is purely voluntary and does not include any monetary refund.

**2.12 Will I (or my Social Security Organization) be charged for this study?**

No, there is no kind of charge or other implication for you or your Social Security Organization for the study.

**2.13 If I agree to participate, how can I learn more information or express any comments on the conducting of the study?**

You can contact the responsible person for the study at any time (Dr Evangelos Kaimakamis – personal telephone number provided).

**2.14 Who will keep this document from the moment I have signed it?**

This document will be filed and kept only by the responsible person for this study (Dr Evangelos Kaimakamis) and will be never displayed to third parties.

**3.  INFORMED CONSENT**

*(This part will be filled by the researcher and the participant)*

**3.1 Identification data of the informed participant in the clinical trial:**

Name of Participant: ________________________________

Date of Birth: _________/_________/_____________

Reference Number: ________________________

**3.2 Consent Form:**

**Study Title:** Study of the respiratory signals of patients with Obstructive Sleep Apnea-Hypopnea Syndrome using complexity theory.

**Place of study:** Sleep Unit of the 2nd Pulmonary Department, General Hospital “G. Papanikolaou” of Thessaloniki and Lab of Medical Informatics, Faculty of Medicine, Aristotle University of Thessaloniki.

|  | **Please circle your choice:** | |
| --- | --- | --- |
| I was informed about the parameters, the aim and the methodology of the study, about potential advantages and disadvantages, as well as potential dangers stemming from it. I was informed orally and in written form by the responsible investigator. | **Yes** | **No** |
| I have read and understood the above information about the study. All my questions were properly addressed. I may keep a copy of my written consent if I wish to do so. | **Yes** | **No** |
| I had enough time to take my decision | **Yes** | **No** |
| I talked with Mr/Mrs.: |  | |
| I understand that I can withdraw from the trial:   - Whenever I wish - Without the obligation to explain my withdrawal - With no effect on my medical treatment | **Yes** | **No** |
| **I agree to take part in the study** | **Yes** | **No** |
| I was given assurance about the confidentiality of my personal data. These data will be fully anonymized in case of future publications of the study results. I accept the fact that local authorities and the scientific council of the hospital may check the data and the methodology of the clinical trial, under the terms of full confidentiality of any personal data, as defined by the law for the protection of personal data secrecy. | **Yes** | **No** |
| If, for any reason, I wish to contact an investigator, I may call Dr Evangelos Kaimakamis, tel. provided. | | |

Signature: ................................................................

Date: .............../................/……………........

Name: ……………..............................................................................

3.3 Declaration of acceptance by a researcher

I provided the potential participant with detailed information about the study, which in my opinion was precise and adequate in order to enable him/her to fully comprehend the nature, the benefits and the potential harms of the trial, as well as the participants’ rights during the trial. There was no external pressure of any kind towards the convincing of the subject to participate. I was present during the signing of the informed consent form by the volunteer.

Researcher’s Name: _________________________________________

Researcher’s Signature: ______________________________________

Date: _______/________/_____________
